# Employment Rate of Newly Certified Healthcare Specialists in Saudi Arabia: A Survey-Based Study

**DOI:** 10.7759/cureus.40898

**Published:** 2023-06-24

**Authors:** Abdulrahman Alesawi, Abdullah Malaka, Maha Abuzenada, Basim Alsaywid, Halla Badawood, Mohammed Aldawsari, Yousif Alshaikh, Norah Alesawi

**Affiliations:** 1 Faculty of Medicine, University of Jeddah, Jeddah, SAU; 2 Research and Development Department, Saudi Commission for Health Specialties, Jeddah, SAU; 3 Department of Occupational Therapy, King Saud Bin Abdulaziz University for Health Sciences, Jeddah, SAU; 4 College of Medicine, King Saud Bin Abdulaziz University for Health Sciences, Jeddah, SAU; 5 Faculty of Medicine, Fakeeh College for Medical Sciences, Jeddah, SAU

**Keywords:** saudi arabia, specialists, dental, medical, employment rate

## Abstract

Background

This study aimed to assess and compare the employment rates of medical and dental specialists across different regions of Saudi Arabia from 2011 to 2019.

Methodology

With the aid of the Saudi Commission for Health Specialties, the national survey was sent via email to 12,000 healthcare postgraduates who obtained their board certificate within the period 2011-2019. It contained several questions regarding demographics, specialty, employment, satisfaction, and, finally, challenges and obstacles they faced during their job-seeking experience.

Results

Of the 723 respondents, almost 655 (90.7%) were employed. The employment rate of medical specialists (n = 605, 90.7%) was higher than dental specialists (n = 50, 89.3%). Nevertheless, 96% (n = 48) of dental employees were working at a specialty of their specified qualification, while only 93.7% (n = 562) of medical employees did. Regarding the month of their employment, the month of October (15.25%) had the highest employment rate for medical specialists; however, for a dental specialist, August (18%) had the highest employment rate. However, 64.5% of the respondents admitted that they faced challenges during their job-seeking experience, with few available positions being the most common obstacle for both medical and dental specialists.

Conclusions

Our survey confirms that medical and dental specialists in Saudi Arabia are facing delays in entering the workforce, which vary by region and specialty. It also sheds light on the reasons for these gaps, with limited job opportunities being a major issue for both groups. For career advancement, it is essential to identify these obstacles and develop a strategy to overcome them, such as involving the private sector.

## Introduction

Medical resident training is a continuing medical education program that aims to develop novice physicians’ capacity to operate autonomously. The quality and development of medical services depend on the training of residents [1]. Workforce planning can be defined as allocating the right people (number and skills) to the right place at the right time to provide the needed services to meet the population’s needs [2,3]. However, failure to do so would result in resource mismanagement, increase the unemployment of skilled personnel, shortage of staff, and decreased quality of the delivered care leading to possible harm to patients [2,3].

It is true that many young professionals, even in distinguished fields, struggle to find work initially, yet this situation is more serious for physicians when jobs are not available in their chosen specialties, as they have fewer alternatives after certification [4]. Moreover, they tend to keep busy in other ways, such as fellowships, locum positions, extra call shifts, and further training in a subspecialty [4]. Based on research conducted in 2012 among internal medicine specialists in the United States, the occupation plan following the internal medicine residency program was reported to be significantly less common than the fellowship program [5]. These results vary depending on the resident’s gender, medical school, region, and program type. However, unemployed residents who are unable to pursue a career in a specific subspecialty are likely to suffer difficulties [5].

According to a survey by the Royal College of Physicians and Surgeons of Canada, around 20% of specialists could not find a job at the time of their certification [4]. Many of these professionals later travel to other countries, such as the United States, to pursue their chosen careers, carrying with them their skills and the government’s investment [4]. Therefore, this study aims to evaluate the employment status of postgraduate specialists across different regions in Saudi Arabia and outline the most common obstacles that they might face during their job-seeking experience.

## Materials and methods

Study design

This is a retrospective, cross-sectional, descriptive study, where a self-administered validated questionnaire was sent through emails to 12,000 Saudi board-certified specialists. The questionnaire contained questions regarding their demographic features, employment status, and several questions about the obstacles they faced during their job-seeking experiences; however, missing data were excluded.

Study eligibility and setting

The inclusion criteria for the study were postgraduate specialists who graduated from medical or dental specialties between 2011 and 2019 and those who accomplished the requirements to finish the Saudi board training inside Saudi Arabia. The sample size was calculated using the Raosoft calculator to be 373 to achieve a confidence interval of 95%, and participants were reached using a convenient non-probability sampling technique. The sample size was raised because it was anticipated that fewer respondents would complete an online survey. The sample was categorized according to their gender, specialties, regions where they were employed, and year of employment.

The questionnaire used in this study consisted of four domains. The first domain aimed to determine the demographic characteristics of the participant. The second domain identified the specific specialty of each participant, and the third domain pinpointed their employment status. The fourth domain aimed to identify the challenges they encountered in achieving their future career goals and plans.

Statistical analysis

SPSS software version 25 (IBM Corp., Armonk, NY, USA) was used for statistical analysis. After being placed in a Microsoft Excel 2010 worksheet, the data were imported into SPSS. Manual thematic analysis with percentages and frequency was utilized to describe the qualitative data. Regarding quantitative data, both mean and standard deviation were used as descriptive analyses for central tendency and variability spread, respectively. The chi-square test was used for inferential analysis. Regarding qualitative data, manual thematic analysis with percentages and frequency was utilized for description. A p-value of less than 0.05 was considered to be statistically significant.

Ethical considerations

This study was approved by the Institutional Review Board of the Saudi Commission for Health Specialties (SCFHS) with the protocol number SRP-000147 in June 2021, and informed consent was mandatory to fill out the questionnaire.

## Results

Demographics

As shown in Table 1, 427 (59.1%) were male, and 296 (40.9%) were female. Regarding regions, Riyadh had the highest number of respondents followed by Makkah region, Eastern province, while Hail region had the least number of respondents. Medical and dental specialists were categorized according to their region; however, the Asir, Qasim, Tabuk, Najran, Al Jawf, Al Baha, Northern Border, and Hail regions were later labeled under “other regions.” Regarding residency graduation year, most respondents were 2019 graduates. Regarding specialty, 667 (92.3%) were medical specialists, and 56 (7.7%) were dentistry specialists. Overall, there were 25 different medical specialties, with general surgeons (n = 97, 14.5%) and family physicians (n = 93, 13.9%) being the most among respondents. On the other hand, there were seven different dental specialties, with endodontics (n = 20, 35,7%) and restorative dentistry (n = 10, 17.9%) specialists having the most responses.

**Table 1:** Demographic characteristics of the respondents.

Variable		N = 723 (%)
Gender	Male	427 (59.1%)
	Female	296 (40.9%)
Year of obtaining the board certificate	2011	34 (4.7%)
	2012	33 (4.6%)
	2013	27 (3.6%)
	2014	49 (6.8%)
	2015	44 (6.1%)
	2016	62 (8.6%)
	2017	75 (10.4%)
	2018	109 (15.1%)
	2019	290 (40.1%)
Specialty	Medicine and surgery	667 (92.3%)
	Dentistry	56 (7.7%)
Region	Makkah	204 (28.2%)
	Riyadh	216 (29.9%)
	Eastern Province	163 (22.5%)
	Asir	24 (3.3%)
	Jizan	13 (1.8%)
	Madinah	42 (5.8%)
	Qasim	22 (3%)
	Tabuk	8 (1.1%)
	Najran	11 (1.5%)
	Al Jawf	3 (0.4%)
	Al Baha	11 (1.5%)
	Northern Border	5 (0.7%)
	Hail	1 (0.1%)

Employment rate

Of the respondents, almost 655 (90.7%) were employed. Generally, the male employment rate (n = 395, 92.5%) was significantly higher than the female employment rate (n = 260, 87.8%) (p = 0.03). The employment rate of medical specialists (n = 605, 90.7%) was higher than dental specialists (n = 50, 89.3%), yet the difference was less than 5% (p = 0.727). However, 96% (n = 48) of dental employees were working at a specialty of their specified qualification, while only 93.7% (n = 562) of medical employees did (p = 0.509). Furthermore, additional analytics were done separately for medicine and dentistry board graduates.

**Figure 1:**
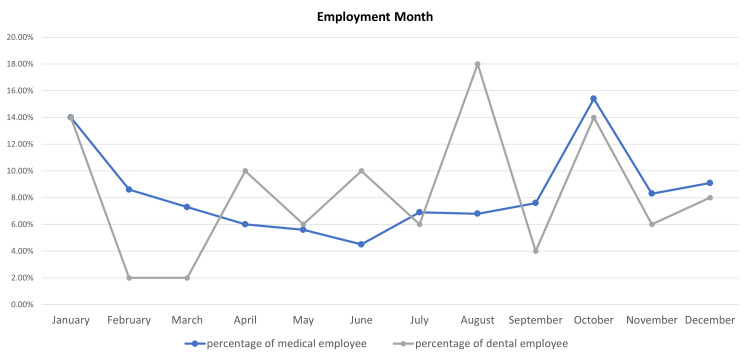
Chart representing the differences in employment rates across different months of the year of both medical and dental specialists.

Regarding the month and year of their employment, October for a total of 10 years (n = 93, 15.4%) and 2020 (n = 149, 24.6%) had the highest employment rate for medical specialties. On the other hand, August for a total of 10 years (n = 9, 18%) and 2011 (n = 16, 32%) had the highest employment rate for dental specialties (Figure 1). Concerning their sector of employment, the Ministry of Health (MOH) employed most dental (n = 38, 76%) and medical specialists (n = 336, 55.5%) (Table 2).

**Table 2:** The employment sector for both medical and dental specialists.

	Employment sector								
	Ministry of Health	Ministry of Defense	Private sector	Ministry of Education/university hospital	Specialist hospital	Ministry of National Guard	Academic position	Ministry of Interior Affairs	Others
Medical specialists (n = 605)	336 (55.5%)	75 (12.4%)	50 (8.3%)	43 (7.1%)	19 (3.1%)	50 (8.3%)	15 (2.5%)	9 (1.5%)	8 (1.3%)
Dental specialists (n = 50)	38 (76%)	4 (8%)	2 (4%)	1 (2%)	0	1 (2%)	3 (6%)	0	1 (2%)

Table 3 illustrates the significant difference in the employment rate of medical specialists in different regions. Regarding medical specialty, the employment rate was highest in “other regions” (n = 81, 100%), followed by Riyadh (n = 183, 91.5%), and the lowest employment rate was in Jizan (n = 11, 84.6%). However, Riyadh (n = 12, 75%), followed by Makkah (n=18, 90%) had the lowest employment rate for the dental specialty.

**Table 3:** Differences in employment rates of medical and dental specialists across different regions in Saudi Arabia. Other regions: Asir, Qasim, Tabuk, Najran, Al Jawf, Al Baha, Northern Border, and Hail.

		Regions						P-value
		Makkah region	Riyadh region	Eastern province	Jizan region	Madinah region	Other regions	
Medical specialists	Employed (n = 605)	159 (86.4%)	183 (91.5%)	141 (91%)	11 (84.6%)	30 (88.2%)	81 (100%)	0.024
	Unemployed (n = 62)	25 (13.6%)	17 (8.5%)	14 (9%)	2 (15.4%)	4 (11.8%)	0	
Dental specialists	Employed (n = 50)	18 (90%)	12 (75%)	8 (100%)	0	8 (100%)	4 (100%)	0.156
	Unemployed (n = 6)	2 (10%)	4 (25%)	0	0	0	0	

**Figure 2:**
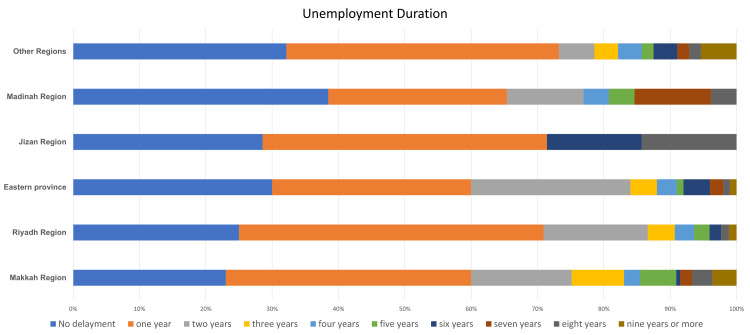
Horizontal stacked bar chart representing the differences in the unemployment duration of different regions. Other regions: Asir, Qasim, Tabuk, Najran, Al Jawf, Al Baha, Northern Border, and Hail. p-value < 0.01.

The periods of unemployment were obtained by calculating the difference from the year of graduation to the year of employment. For medical specialists in different regions, the unemployment duration showed an average of one-year delay as the highest percentage for all regions with an exception in the Madinah region, where it showed no delay in the employment rate for most participants. For dental specialists, the total average unemployment duration showed no delay as the most common in all regions. However, most Makkah specialists had waited for five or more years to get a job (Figure 2).

**Figure 3:**
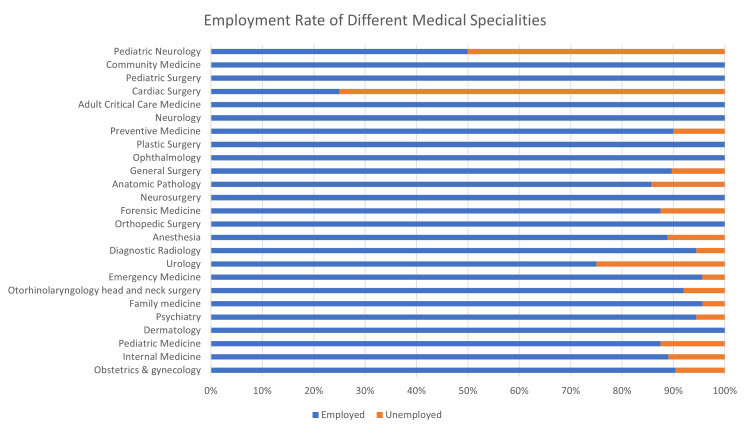
Horizontal stacked bar chart representing the differences in employment rates of different medical specialties. p-value = 0.01.

Medical specialties had significant differences in employment rates, with dermatology, community medicine, adult critical care medicine, pediatric surgery, plastic, neurology, ophthalmology, neurosurgery, and orthopedic surgery having the best employment rate among them (100%) (Figure 3).

Counseling

Only eight (5.8%) of the 138 medical specialists who received adequate counseling were unemployed. However, out of the 529 medical specialists who did not receive adequate counseling, 54 (10.2%) were unemployed (p = 0.11). More than half (n = 383, 52.2%) of the respondents stated that they would not change their specialty even if they could see the needed specialty via a dashboard before engaging in the program.

Employment challenges

**Figure 4:**
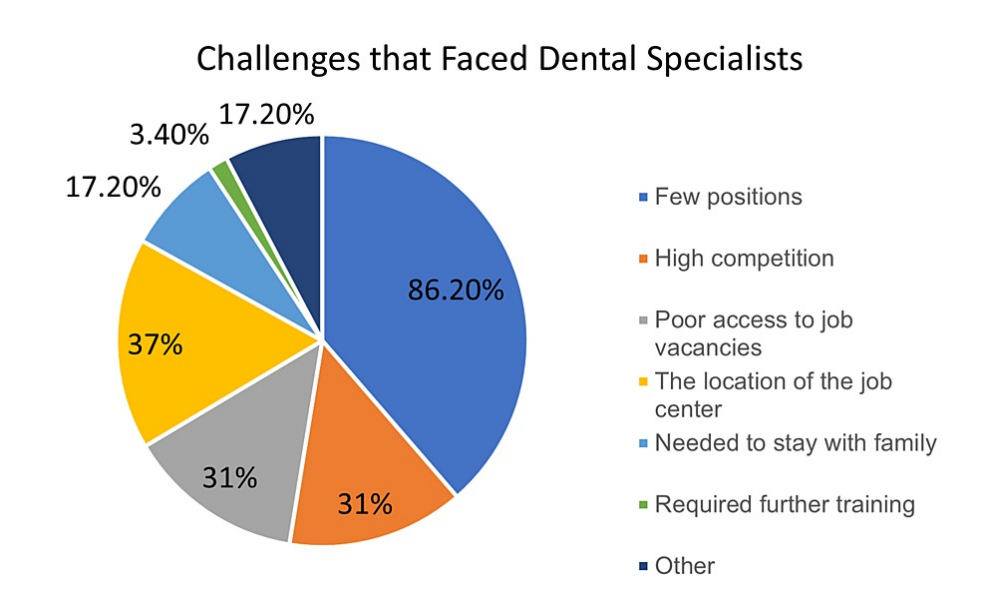
Pie chart representing the challenges faced by dental specialists during their job-seeking experience.

**Figure 5:**
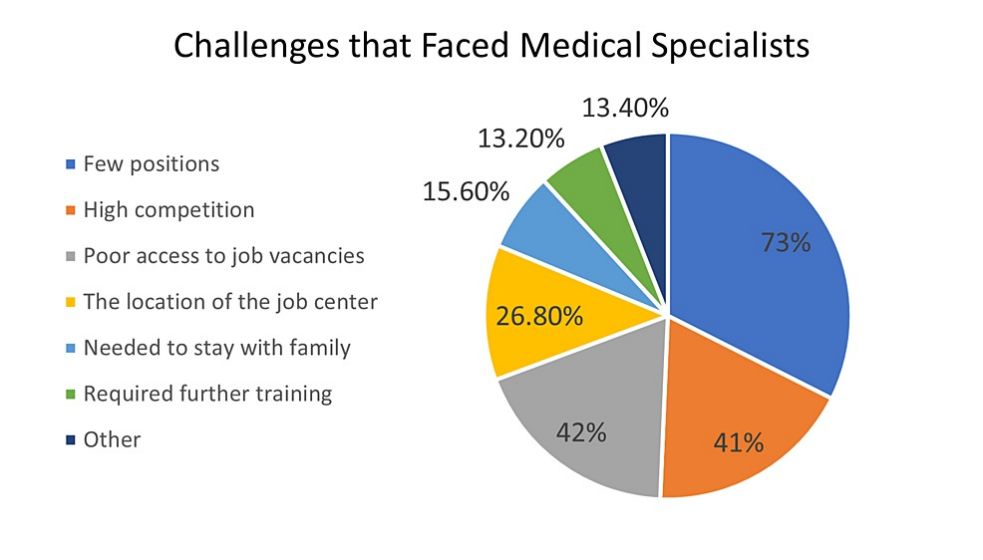
Pie chart representing the challenges faced by medical specialists during their job-seeking experience.

In general, 64.5% of the respondents admitted that they faced challenges during their job search. In the questionnaire, several challenges were listed as options and they were able to choose however many they encountered. A few positions were the most common complaint for both medical (73%) and dental (86.2%) specialists. A few of the medical (13.2%) and dental (3.4%) specialists illustrated that they would need further training to get a job (Figures 4, 5).

Satisfaction and future plans

Employment seeking outside Saudi Arabia for both medical and dental specialists who were satisfied with their employment (n = 328, 49.69%) was less when compared to those who were not satisfied with their employment (n = 332, 50.31%). Moreover, seeking employment outside Saudi Arabia was also higher when they were satisfied with their salary (n = 271, 41.06%) compared to those who were not (n = 389, 58.94%) (p < 0.01). Furthermore, the results illustrated that approximately 24-25% of those who were not satisfied with their employment/salary were seeking jobs outside Saudi Arabia.

Recommended solutions

A total of 252 responses to an open-ended short-answer question regarding the recommended solution to improve employment are summarized into five themes in Table 4, which are (1) encouraging the private sector into participating, (2) having a common unbiased method of assessment for healthcare providers in all healthcare centers, (3) offering a different kind of support to residents through a collaborative relationship between the SCFHS and the MOH, (4) running more hospitals and offering more positions, and (5) healthcare centers should be running independently of MOH.

**Table 4:** Recommended solutions by respondents for unemployment/delayed employment.

Themes	Responses
1. Encouraging the private sector into participating	“Regulation for minimum salary in the private sector,” and “Use of one portal for all private vacancies”
2. Having a common unbiased method of assessment for healthcare providers in all healthcare centers	“Need for administrative support.” Need to remove unprofessional behavior and biased reports.” “Need for patient-oriented health services and following equal rules and regulations for caregivers.” “To be united on the website for all MOH and non-MOH and even private, making all application online”
3. Offering a different kind of support to residents through a collaborative relationship between the SCFHS and MOH	“Show access to employers by advertising opportunities to graduates.” “Clear announcement from the SCFHS about available vacancies everywhere in our country or gulf region.” “An official website for SCHSS graduate doctors with their qualification and contact methods for easy search by institutes”
4. Running more hospitals and offering more positions	“Increase the number of positions to accommodate the need of the patients. A lot of hospitals are understaffed and pressured by high volume of patients.” “Build more hospital centers, availability, simplicity of job applicants”
5. Healthcare centers should be running independently of the MOH	“System is bad, especially with how people manage hospitals and all the MOH hospitals should be self running independent of the ministry.” “To enforce training programs, to hire their own graduated residents for one year as a specialist after graduation to help these graduates to gain better chance of employment in other places”

## Discussion

Employment rate and challenges

Utilizing a census of all medical and dental residency program graduates in Saudi Arabia between 2011 and 2019, we find that almost 10% of medical and dental specialists are unemployed. However, it is considered to be better when compared to the unemployment rate of Saudi Arabia’s general workforce among citizens from 2016 to 2020 which was established in 2020, as it ranged from 11.6% to 15.4% [6].

The problem of doctors’ employment is not limited to a single country. The unemployment rate was lower among master’s degree graduates in 2016 in the United States (2.4%) [7]. Italy with a 17% unemployment rate among doctors in 1990, Austria with a rate of 9%, Germany with 8%, the Netherlands with 6%, and Spain with 5% are among the European countries that have battled with the problem of unemployed doctors [8]. Another study conducted in the United States showed that 67.3% of the resident physicians who applied for jobs got clinical practice jobs in their specialties, 15.5% got academic jobs, 5.0% got clinical jobs in other specialties, and 5.1% had other plans [9].

This study evaluated the employment rate of medical specialties in different regions and suggests that low-population regions have higher employment rates than regions with high populations. However, the fact that residents consider spending time with their family as the most important factor determining their career choices explains why most physicians have the intention to practice in large towns despite their knowledge that opportunities mostly exist in rural areas [10,11].

Regarding medical specialties, there is an observed relationship in surgical specialties regarding employment, as the employment rate was more than 90% in most surgical specialties, with cardiac surgery, general surgery, and urology being an exception. These findings might be explained by the high career satisfaction rate among surgical residents [12].

As unemployment rises, those impacted may be at a higher risk of developing or aggravating mental and physical health issues and will have less life satisfaction that persists for a long time [13,14]. A study that was conducted during the COVID-19 pandemic found that individuals who were not working due to out-of-hand reasons during the pandemic had a higher prevalence of mental health problems than those who were working and those who freely left work [15].

It is possible that unemployment might be due to many different causes, but the four main reasons that were found among master’s degree graduates are (1) few or no jobs for the specified qualifications, (2) the jobs offered are not preferred, (3) being unstable and relocating between jobs, and (4) preference of being unemployed at the time [7].

Recommended solutions

Decreased or delayed employment rate is a problem that is subject to solutions, and some of these solutions were offered by the participants. Including private sector participation and having an unbiased shared method of assessment among all healthcare centers. Moreover, decreasing working hours per physician is a solution that provides extra hours for other unemployed physicians; however, the disadvantage of less clinical exposure for each physician may be a possibility [16]. A study conducted by Wakeam et al. recommended practice sharing as a solution for Canadian surgeons. This concept recommends senior surgeons partner with young surgeons just starting as they wind down their practices [17].

Some respondents suggested promoting hospitals in peripheral cities by providing high standards or a raise in the salary to occupy the need in the job market. Alluhaidan et al. [18] stated that the current number of fellowships or board positions available is inadequate to accommodate all graduates from medical schools, which is one of the most common responses we received. It was also suggested that the MOH and SCFHS align and collaborate to balance the candidates and offer training positions that match the available job vacancies.

For vocational improvement, many respondents reported that there is a need for improving salaries and increasing recruitment; specifically, offering more job opportunities both in the governmental and private sectors. As described in 2019, a study mentioned that there are several workforce-related challenges such as payment. Payment is not associated with performance, which leaves physicians with little motivation to work [18]. Another study suggested that future unemployment among doctors could be remedied by restricting admission to medical school [8]. Some other ideas such as workforce planning, increased funding, more creative use of resources, and running operating rooms on weekends have been proposed to improve the employment condition for specialists [4]. Moreover, residents may require additional business-related training, which might come from attendings, local attorneys, office managers, and former residents [19].

Limitations of the study

To our knowledge, this study is the first to evaluate the employment rate among medical and dental specialists in Saudi Arabia. However, as with other cross-sectional studies, it is subject to limitations. Low response rates, recall biases, and selection biases are some of the downsides. Thus, further studies are recommended to be performed utilizing different methodologies.

## Conclusions

Our questionnaire verified that medical and dental specialists in Saudi Arabia are encountering delayed entry to the workforce, which is diverting in different regions and specialties. Moreover, it provided a brief insight into the causes of these employment gaps, with few available positions being the most common reason among both dental and medical specialists. Nevertheless, more work must be done to explore the challenges of each specialty. For career advancement, these obstacles should be highlighted, and a plan to overcome the challenges in the healthcare workforce such as encouraging private sectors into participating and setting common unbiased standards of evaluation should be proposed.
